# Incubation Period for Human Cases of Avian Influenza A (H5N1) Infection, China

**DOI:** 10.3201/eid1411.080509

**Published:** 2008-11

**Authors:** Yang Huai, Nijuan Xiang, Lei Zhou, Luzhao Feng, Zhibin Peng, Robert S. Chapman, Timothy M. Uyeki, Hongjie Yu

**Affiliations:** Chinese Center for Disease Control and Prevention, Beijing, People’s Republic of China (Y. Huai, N. Xiang, L. Zhou, L. Feng, Z. Peng, H. Yu); Chulalongkorn University, Bangkok, Thailand (Y. Huai, R.S. Chapman); Centers for Disease Control and Prevention, Atlanta, Georgia, USA (T.M. Uyeki)

**Keywords:** incubation period, avian influenza (H5N1), China, letter

**To the Editor:** Since 1997, more than 400 human cases of highly pathogenic influenza A virus (H5N1) infection have been reported worldwide, including 30 from mainland China. Ascertainment of the incubation period for influenza virus (H5N1) is important to define exposure periods for surveillance of patients with suspected influenza virus (H5N1) infection. Limited data on the incubation period suggest that illness onset occurs <7 days after the last exposure to sick or dead poultry (*1*–*4*). For clusters in which limited human-to-human virus transmission likely occurred, the incubation period appeared to be 3–5 days (*5*–*7*) but was estimated to be 8–9 days in 1 cluster (*5*). In China, exposure to sick or dead poultry in rural areas and visiting a live poultry market in urban areas were identified as sources of influenza A virus (H5N1) exposures (*8*), but the incubation period after such exposures has not been well described.

We conducted a retrospective descriptive study of 24 of 30 influenza virus (H5N1) cases in China to estimate and compare incubation periods for different exposure settings, including case-patients exposed only to sick or dead poultry versus those exposed only to a wet poultry market, where small animals and poultry may be purchased live or slaughtered (www.searo.who.int/en/Section23/Section1001/Section1110_11528.htm). Exposures may be direct (e.g., touching poultry) or indirect (e.g., no physical contact, but in close proximity to poultry, poultry products, or poultry feces). We excluded 6 cases, including 2 with unavailable epidemiologic data, 1 without an identified exposure source, 2 in a cluster with limited person-to-person transmission (*6*), and 1 in which the patient was exposed to both a wet poultry market and to sick or dead poultry. Epidemiologic data were collected through patients and family interviews and a review of case-patients’ medical records.

The incubation period was defined as the time from exposure to symptom onset. The maximum time from first exposure to illness onset was limited to 14 days for biological plausibility. For case-patients with exposures on multiple days, we calculated each case-patient’s median incubation period and then calculated the overall median and range of the distribution of these median incubation periods. Similarly, the minimum and maximum incubation periods for case-patients with exposures on multiple days was estimated by using the last or first known exposure day, respectively. The overall incubation period among these case-patients was estimated by determining the median of the distribution of case-patients’ median incubation periods. Incubation periods were compared by using the Wilcoxon rank-sum test. All statistical tests were 2-sided with a significance level of α = 0.05.

Of the 24 case-patients, 16 (67%) had exposure to sick or dead poultry only (median age = 25 years [range 6–44]; 25% male; 100% lived in rural areas). Eight (33%) had visited a wet poultry market only (median age = 30 years [range 16–41]; 63% male; 88% [7/8] lived in urban areas) (Table). For case-patients with >2 exposure days (n = 18), and for case-patients with a single exposure day (n = 6), the overall median incubation period was longer for those who had visited a wet poultry market than for those who were exposed to sick or dead poultry, but the difference was not significant. When data for single and multiple exposure days were combined, the overall median incubation period for case-patients exposed to a wet poultry market (n = 8) was significantly longer than for case-patients (n = 16) exposed to sick or dead poultry (7 days [range 3.5–9] vs. 4.3 days [range 2–9]; p = 0.045).

**Table:** Estimated incubation period of 24 human cases of infection with avian influenza A virus (H5N1), China*

Exposure data	Case-patients with exposure to sick/ dead poultry only	Case-patients with exposure to wet poultry market only	p value	All case-patients
No. case-patients with exposures on multiple days	12	6		18
Overall median incubation period, d (range)	4.5 (2–9.5)	6.3 (3.5–7)	0.276	5 (2–9.5)
Median of minimum incubation period, d (range)	1 (0–5)	0 (0–2)	0.315	0.5 (0–5)
Median of maximum incubation period, d (range)	7.5 (4–14)	11.5 (7–14)	0.108	8.5 (4–14)
No. case-patients with single known exposure	4	2		6
Overall median incubation period, d (range)	3.5 (2–6)	8.5 (8–9)	0.064	5 (2–9)
All case-patients	16	8		24
Overall median incubation period, d (range)	4.3 (2–9)	7 (3.5–9)	**0.045**	5 (2–9.5)
Overall median of minimum incubation period, d (range)	1.5 (0–6)	1 (0–9)	0.752	1.5 (0–9)
Overall median of maximum incubation period, d (range)	6 (2–14)	9 (7–14)	**0.031**	7.5 (2–14)

*Boldface represents significant results (Wilcoxon rank-sum test).

Our findings are subject to limitations. Proxies for deceased case-patients may not have known all of the case-patient’s exposures. Surviving case-patients may not have recalled or identified all exposures that occurred, including environmental exposures. It was impossible to ascertain when infection occurred for case-patients with multiple days of exposures. Our limited data did not permit the use of other methods such as survival analysis to better define incubation periods. We did not quantify exposure duration and could not determine whether repeated exposures (dose-response) or a threshold of exposure to influenza virus (H5N1) exists to initiate infection of the respiratory tract. Laboratory testing was not performed to confirm that the exposure sources contained influenza virus (H5N1) or to quantify exposures.

Despite exposures of many persons in China to sick or dead poultry or to wet poultry markets, human influenza A (H5N1) disease remains very rare. Our findings suggest that the incubation period may be longer after exposure to a wet poultry market than after exposure to sick or dead poultry, and, therefore, a longer incubation period than the 7 days that is used widely (*4*,*9*) could be considered for surveillance purposes. However, because of the small number of influenza virus (H5N1) case-patients, our study was too underpowered to draw any firm conclusions; results should be interpreted cautiously. In a study of influenza virus (H5N1) cases in Vietnam, 5 case-patients did not have any identified exposure <7 days of illness onset (*10*). In China, the exposure period for surveillance of suspected influenza virus (H5N1) cases now includes exposure to a wet poultry market <14 days before illness onset. Although data on person-to-person virus transmission are limited, close contacts of patients infected with influenza virus (H5N1) in China are monitored daily for 10 days after the last known exposure. Further studies are needed to quantify the incubation period after exposure to sick or dead infected poultry, a wet poultry market, or to an influenza A virus (H5N1) case-patient and to investigate the basis for any differences.
